# Antimycobacterial Activity, Synergism, and Mechanism of Action Evaluation of Novel Polycyclic Amines against *Mycobacterium tuberculosis*

**DOI:** 10.1155/2021/5583342

**Published:** 2021-06-11

**Authors:** Erika Kapp, Jacques Joubert, Samantha L. Sampson, Digby F. Warner, Ronnett Seldon, Audrey Jordaan, Margaretha de Vos, Rajan Sharma, Sarel F. Malan

**Affiliations:** ^1^School of Pharmacy, Faculty of Natural Sciences, University of the Western Cape, Cape Town, South Africa; ^2^DSI/NRF Centre of Excellence for Biomedical Tuberculosis Research, SAMRC Centre for Tuberculosis Research, Division of Molecular Biology and Human Genetics, Faculty of Medicine and Health Sciences, University of Stellenbosch, Cape Town, South Africa; ^3^SAMRC/NHLS/UCT Molecular Mycobacteriology Research Unit, DSI/NRF Centre of Excellence for Biomedical Tuberculosis Research, Department of Pathology and Institute of Infectious Disease and Molecular Medicine, Faculty of Health Sciences, University of Cape Town, Cape Town, South Africa; ^4^H3D Drug Discovery and Development Centre, Department of Chemistry, University of Cape Town, Cape Town, South Africa

## Abstract

*Mycobacterium tuberculosis* has developed extensive resistance to numerous antimycobacterial agents used in the treatment of tuberculosis. Insufficient intracellular accumulation of active moieties allows for selective survival of mycobacteria with drug resistance mutations and accordingly promotes the development of microbial drug resistance. Discovery of compounds with new mechanisms of action and physicochemical properties that promote intracellular accumulation, or compounds that act synergistically with other antimycobacterial drugs, has the potential to reduce and prevent further drug resistance. To this end, antimycobacterial activity, mechanism of action, and synergism in combination therapy were investigated for a series of polycyclic amine derivatives. Compound selection was based on the presence of moieties with possible antimycobacterial activity, the inclusion of bulky lipophilic carriers to promote intracellular accumulation, and previously demonstrated bioactivity that potentially support inhibition of efflux pump activity. The most potent antimycobacterial demonstrated a minimum inhibitory concentration (MIC_99_) of 9.6 *μ*M against *Mycobacterium tuberculosis* H37Rv. Genotoxicity and inhibition of the cytochrome *bc*_*1*_ respiratory complex were excluded as mechanisms of action for all compounds. Inhibition of cell wall synthesis was identified as a likely mechanism of action for the two most active compounds (**14** and **15**). Compounds **5** and **6** demonstrated synergistic activity with the known Rv1258c efflux pump substrate, spectinomycin, pointing to possible efflux pump inhibition. For this series, the nature of the side chain, rather than the type of polycyclic carrier, seems to play a determining role in the antimycobacterial activity and cytotoxicity of the compounds. Contrariwise, the nature of the polycyclic carrier, particularly the azapentacycloundecane cage, appears to promote synergistic activity. Results point to the possibility of combining an azapentacycloundecane carrier with a side chain that promotes antimycobacterial activity to develop dual acting molecules for the treatment of *Mycobacterium tuberculosis.*

## 1. Introduction

The progressive development of resistance to various chemotherapeutic agents used in the management of infectious diseases presents a serious problem in global public health. Tuberculosis (TB) has re-emerged as one of the most concerning communicable diseases of our time. The notoriously complex structure of the mycobacterial cell wall and the abundance of efflux pumps (EPs) in *Mycobacterium tuberculosis* limit the intracellular accumulation and antimycobacterial efficacy of numerous antimicrobial classes successfully utilized against standard bacteria [1–4]. Treatment and control of the TB epidemic is further complicated by the development of multidrug-resistant (MDR) and extensively drug-resistant (XDR) strains. Current TB treatment regimens thus comprise a combination of antimycobacterial agents which, as drug resistance develops to the various first-line agents, not only decrease in efficacy and patient acceptability, but also increase in required treatment duration and toxicity.

Based on their mechanisms of action (MOAs), available antimycobacterial drugs can be broadly categorized into three classes: those which disrupt cell wall integrity (e.g., isoniazid, ethambutol, ethionamide, and cycloserine, as well as the experimental 1,2-ethylenediamine, SQ109 [5]); those that limit the energy available for cellular processes (e.g., pyrazinamide and bedaquiline [6]); and compounds that inhibit normal cellular functionality by inhibiting or corrupting biosynthesis of essential macromolecules, cofactors, and metabolites (e.g., the rifamycin, fluoroquinolone, aminoglycoside and oxazolidinone class antibiotics, and para-aminosalicylic acid [7–9]). Reduced susceptibility to these antimycobacterial agents commonly results from spontaneous mutations in the genome of mycobacteria with selective survival fueled by suboptimal exposure to, or reduced intracellular accumulation of, active moieties [10, 11]. EPs, in particular, have been shown to play a role in inadequate accumulation of drugs within the mycobacterial cell [12]. Depending on the particular mechanism by which a drug's action is overcome, resistance may be specific to a particular molecule or, more alarmingly, result in reduced sensitivity to various drugs that target a particular cellular process (e.g., cell wall synthesis) or act as substrates for an overexpressed efflux pump.

Strategies that improve the accumulation of antimycobacterial compounds within the mycobacterial cell would likely increase the efficacy of the particular compound, but would also minimize genetically encoded resistance aided by subinhibitory chemotherapeutic exposure [13]. Approaches that may promote intracellular accumulation of chemotherapeutic agents could include increasing cell wall permeability [14], facilitation of passive transport of drugs into the cell, alteration of the chemical structure of molecules to reduce their predisposition to efflux [15], as well as direct efflux pump inhibition through the utilization of EP inhibitors [16].

**Figure 1:**
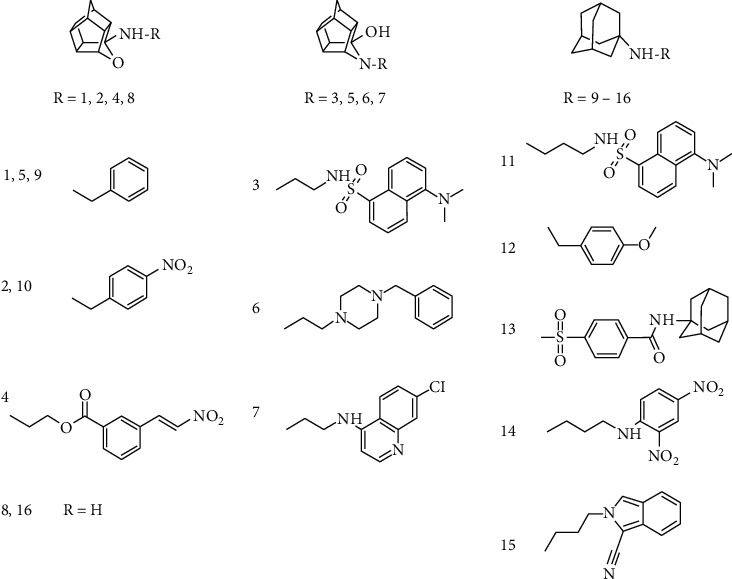
Structures of evaluated compounds grouped according to the respective lipophilic scaffold.

As part of a research project designed to evaluate the possibility of modulating drug resistance and increase accumulation of active moieties in *M. tuberculosis*, a series of polycyclic cage compounds were selected from the University of the Western Cape School of Pharmacy compound library to be evaluated for antimycobacterial and efflux pump inhibitory activity in *M. tuberculosis.* The polycyclic cage derivatives included in this study demonstrate, amongst others, *L*-type calcium channel and *N*-methyl-D-Aspartate (NMDA) inhibitory properties [17] and feature a lipophilic scaffold likely to improve the barrier permeability of selected structures. Subsequent research demonstrated the ability of polycyclic amine derivatives (particularly compounds **3**, **5**, and **11**, Figure 1) to modulate antimalarial drug resistance [18–20]. Criteria for the selection of compounds to include in this study were therefore the presence of moieties with possible antimycobacterial activity, and the inclusion of bulky lipophilic carriers to promote compound accumulation within the mycobacterial cell, as well as biological effects which may promote resistance reversal activity. The possession of potential ion channel inhibitory properties, which may directly or indirectly inhibit efflux pump efficacy [21, 22], was an important consideration in compound selection.

Here, we report the antimycobacterial activities of the series of selected compounds and a preliminary investigation into possible MOAs of the active molecules. We also describe a possible modulation of Rv1258c efflux pump activity as indicated by synergistic activity with spectinomycin, a known substrate for the mycobacterial Rv1258c efflux pump [15].

## 2. Results and Discussion

The compounds selected for the study were classified into three categories based on the nature of the polycyclic cage scaffold. Figure 1 shows the oxapentacycloundecane class (compounds **1**, **2**, **4**, and **8**), the azapentacycloundecane class (compounds **3**, **5**, **6**, and **7**), and adamantane class (compounds **9–16**). Synthesis of compounds **1**–**15** (Figure 1) is described in detail in previous publications by our group, as indicated in Table 1. Compound **16** was purchased from Sigma-Aldrich®.

### 2.1. Antimycobacterial Activity

Minimum inhibitory concentrations (MIC_99_) of the compounds (Table 1) were determined in nutrient-rich and nutrient-poor media types by the broth microdilution method utilizing an *M. tuberculosis* H37Rv reporter strain expressing GFP [29–31]. Compounds **3, 5, 7, 11, 14**, and **15** showed antimycobacterial activity with MIC_99_ values ranging from 9.6 *μ*M to 82.2 µM (Table 1); these were selected for further analysis to determine the possible MOA.

Interestingly, during MIC determination, compound **5** showed activity in the GAST-Fe minimal medium, comprising glycerol, alanine, salts, iron, and Tween80, but not in the standard Middlebrook 7H9 albumin-dextrose-catalase (ADC) medium. This difference may point to the binding of the compound by albumin [22], interference of catalase in the MOA, or an MOA that has an impact on the ability of the bacillus to use glycerol as the primary carbon source [32]. In contrast, the activities of compounds **7** and **14** were much more pronounced in standard 7H9-ADC, while compounds **3, 11**, and **15** showed comparable potencies in both media types.

**Figure 2:**
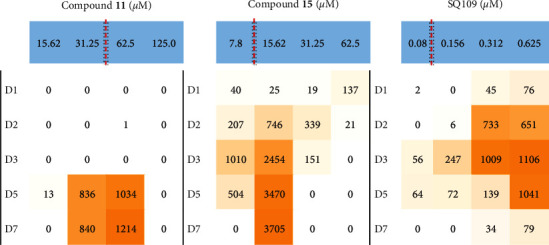
Observed luminescence data of compounds **11**, **15**, and SQ109 depicting cell wall damage over 7 days (D1–D7) at various concentration ranges (*μ*M) spanning the MIC_90_ (as indicated by the red dotted line).

An interesting observation pertaining to the structures of the compounds versus the observed antimycobacterial activity was the differences in MIC for the structurally similar compound **1**, an oxapentacycloundecane benzyl amine derivative, and the azapentacycloundecane benzyl amine derivative, compound **5**. Results point towards antimycobacterial activity being linked to the presence of the tertiary amine and/or free hydroxyl group within the polycyclic aza-cage. Compounds **11**, **14**, and **15** containing an adamantane moiety showed better activity compared to oxa- and aza-PCU-based compounds in general. These are also the only 3 compounds containing the 1,3-diamine linker *versus* the 1,2-diamine present in compounds **3, 6,** and **7**. This increased activity could, therefore, be due to the presence of the adamantane or the 1,3-diamine linker. Comparable activity (Figure 2) seen in compounds **2** (oxa-PCU) and **10** (adamantane derivative), however, suggest that the increased activity may be linked to the 1,3-diamine linker rather than the presence of the adamantane moiety.

### 2.2. Cytotoxicity Analysis

Cytotoxicity of the compounds was evaluated using a Chinese hamster ovarian (CHO) cell line (Table 1). The marked cytotoxicity differences observed between compounds **3** and **11** were noteworthy. The adamantane moiety may contribute to increased cytotoxicity, but, as can be deduced from the similar IC_50_ values of compounds **2** and **10**, it appears that this molecular feature cannot be solely responsible for the increased cytotoxicity observed for compound **11**. The propane-1,3-diamine linker, however, seems to contribute to cytotoxicity as compounds **11**, **14**, and **15** all demonstrate significantly higher cytotoxicity, even when compared to most compounds containing the similar ethane-1,2-diamine moiety. The 1,3-diamine linker unfortunately also seems to play a role in antimycobacterial activity as these compounds demonstrate the lowest overall MIC values.

### 2.3. Preliminary Mechanism of Action Determination

To explore the potential MOA of the compounds, a number of bioactivity reporter assays were utilized. To evaluate the possible impact of the compounds on respiration, comparative MIC values were determined for the respective compounds in wild-type *M. tuberculosis* H37Rv vs. (i) a cytochrome *bd* oxidase deficient mutant (∆*cyd*KO) and (ii) a ∆*cyd*KO*/qcrB*A317T strain to determine possible QcrB inhibition [33, 34]. QcrB inhibitors are expected to exhibit increased potency (lower MIC) in the ∆*cyd*KO but minimal activity in the ∆*cyd*KO*/qcrB*A317T strain which carries an additional Ala317Thr point mutation in QcrB [35]. No significant shift in MIC was observed for any of the compounds against either of the mutant strains, strongly suggesting that none was likely to target QcrB [34, 36].

Next, a real-time bioluminescence assay [37] was used to investigate cell wall damage as a potential mechanism of action. Compounds with inhibitory effects on mycobacterial arabinogalactan, mycolic acid, and fatty acid synthesis have been shown to result in the upregulation of the *iniBAC* operon [38]. This operon is, however, not upregulated by other cell wall stressors, for example, exposure to hydrogen peroxide, heat, acidic conditions, or antibiotics that do not directly inhibit cell wall synthesis (e.g., aminoglycosides, fluoroquinolones, or rifampins) [38]. Compounds **14** and **15** were P*iniB-*LUX positive and showed early (day 1) and sustained luminescence signals, similar to the luminescence profile which was observed previously for ethambutol [37]. Figure 2 provides an example of bioluminescence observed for late and indirect cell wall damage (compound **11**), early cell wall damage (compound **15**), and the positive control SQ109 (also demonstrating early cell wall damage). Comparative background-corrected RLU data are presented as heat maps, ranging from white (minimum) to orange (maximum) as a function of the maximum RLU recorded for luminescence control.

Given the presence of the adamantane and amine linker functional groups in SQ109 [39], it was decided to use SQ109 as a control compound in the P*iniB-*LUX assay. SQ109 also demonstrated early luminescence, but the signal was only sustained for approximately 5 days [37], whereas in the current study, a continued signal was observed for compounds **14** and **15** over the course of the 14-day assay. Compounds **3**, **7**, and **11** were associated with delayed production of bioluminescence. This suggests that the impact on cell wall homeostasis might be delayed or a secondary effect of a compound with polypharmacologic activity. These data suggest that compounds **14** and **15** directly target cell wall biosynthesis, although the precise target is still unknown.

Finally, we investigated whether any of the compounds were genotoxic using the P*recA-*LUX bioluminescence reporter [37]. RecA is a key regulator of mycobacterial DNA damage response and is induced after the exposure to compounds which are directly genotoxic (i.e., altering or damaging to the nucleic acid) or which inhibit DNA metabolism (replication and/or repair). Placing the bacterial luciferase *luxCABE* cassette under the transcriptional control of the *recA* promoter results in increased bioluminescence following promoter induction in response to DNA damage [37]. None of the compounds evaluated in this study produced a positive luminescence signal over the full duration of the assay, eliminating DNA damage as potential MOA.

### 2.4. Synergism Evaluations

**Figure 3:**
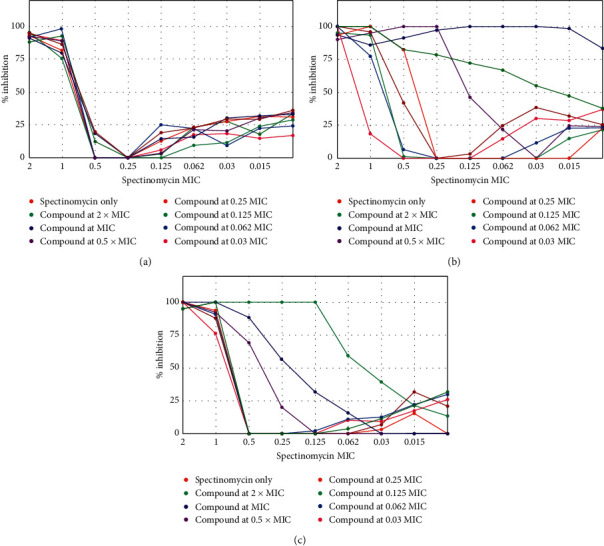
Percentage inhibition of compound **1** (a), **5** (b), and **6** (c) at various concentration combination points where the concentration of spectinomycin (as a function of MIC) is indicated on the *x*-axis combined with multiples of the MIC of the test compound as indicated by the colors in the legend.

It was originally postulated that compounds from this series might act as mycobacterial efflux pump inhibitors (EPIs). As an initial screen to determine possible EPI activity, synergism assays were performed using the known Rv1258c efflux pump substrate, spectinomycin, as an anchor compound. Spectinomycin is an aminocyclitol antibiotic with a unique binding site on the bacterial 30S ribosomal subunit which affords it selective toxicity and a good side effect profile. Unfortunately, mainly due to extensive efflux by bacterial efflux pumps (specifically Rv1258c in *M. tuberculosis*), spectinomycin lacks significant antibacterial activity [15, 40, 41]. The activity of spectinomycin is increased substantially under conditions where efflux pump activity is inhibited [42] making it a useful agent to screen for possible efflux pump inhibitory activity using synergism assays. All compounds apart from compounds **14** and **15**, which were excluded due to inherent antimycobacterial activity, were screened for synergism with spectinomycin using a checkerboard assay. To this end, serial dilutions of the test compound and spectinomycin were added to an *M. tuberculosis* culture in 7H9 media on the *y*- and *x*-axis of a 96-well plate. Bacterial growth was measured, and data were analyzed as described [43]. Compounds **5** and **6** showed evidence of synergism with spectinomycin, but no significant changes in MIC were observed with any of the other compounds. Inhibition of bacterial growth for compounds **1**, **5**, and **6** in combination with spectinomycin is shown in Figure 3. The concentration of spectinomycin as a fraction of its MIC (i.e., 2 = 2 × MIC, 1 = MIC, 0.5 = half MIC etc.) is given on the *x*-axis, combined with various fractions of the MIC of the test compound as indicated by the colors in the graph legend. The bacterial inhibition as measured in the spectinomycin-only wells at various multiples of spectinomycin MIC is depicted as the dark red (top) legend on the graph. If spectinomycin is combined with a test compound with which it synergizes, the percentage bacterial inhibition remains high despite decreases in spectinomycin concentration. Thus, compound **1** (A) which is provided for purposes of comparison with structurally similar compound **5** shows no evidence of synergism, but bacterial inhibition at lower concentrations of spectinomycin when combined with compounds **5** (B) and **6** (C) point to synergistic activity.

Both compound **5** and **6** are aza-cage derivatives. Again, the differences between the activity of the structurally similar compounds **1** and **5** (Figure 1) are notable. Similar to what was seen with the antimycobacterial activity of these two compounds, the tertiary nitrogen and free hydroxyl group on the aza-cage are likely involved in the observed synergistic activity. Compounds **3** and **7**, contrariwise, appeared to increase the MIC of spectinomycin at higher concentrations (data not shown). This observation might point to reduced absorption of spectinomycin but would require further investigation to determine the mechanism of this possible antagonism.

## 3. Conclusions

In this study, we explored possible antimycobacterial activity, mechanism of action, and synergistic activity of a series of polycyclic amine derivatives. The compounds were selected based on the presence of pharmacophoric moieties with possible antimycobacterial activity, previously described resistance reversal activity, as well as for the presence of structural features and bioactivity that may increase the accumulation of active moieties and coadministered antimycobacterial drugs within *M. tuberculosis*. The most active compound (**15**) demonstrated an MIC_99_ of 9.6 *μ*M against *M. tuberculosis* H37Rv and will serve as a lead compound to improve selective antimycobacterial activity.

Inhibition of the *bc*_*1*_ respiratory complex and DNA functionality as MOA as well as genotoxicity were excluded for the series. Cell wall damage, however, was identified as the likely mechanism of action of a number of compounds. Particularly, compounds **14** and **15** were shown to cause early and sustained cell wall damage in a fashion similar to ethambutol. Compounds **3**, **7**, and **11** showed delayed cell wall damage which may indicate cell wall stress as a secondary effect to interference with other cell systems or a delayed effect on cell wall integrity.

Compounds **5** and **6** showed evidence of synergistic activity with spectinomycin and thus possible efflux pump (specifically Rv1258c) inhibition.

The azapentacycloundecane cage seems to play a role in the antimycobacterial activity in nutrient poor media as well as the ability to synergize with spectinomycin as is evident from the large variances between the structurally similar compounds **1** and **5** in the respective assays. The role of the aza-cage in antimycobacterial and synergistic activity thus warrants further exploration. Although compounds from this series do not display sufficiently selective toxicity in single-drug *in vitro* assays, the ability of selected compounds from this series to act as chemosensitizers, may reduce the required MIC in combination therapy. Results from this preliminary study have contributed to our understanding of the structure activity relationships, possible synergistic activity, and toxicity for this series of compounds and will be used in conjunction with ongoing synergism assays to inform lead modification. Additional work exploring structure activity relationships for antimycobacterial activity (versus cytotoxicity) and, importantly, investigations pertaining to possible resistance reversal activity are underway in our laboratories.

## 4. Materials and Methodology

### 4.1. Minimum Inhibitory Concentration Determination

All assays were performed in a Biosafety Level III certified facility. *Mycobacterium tuberculosis* pMSp12:GFP [29, 44] was grown to an optical density (OD) of 0.6–0.7, and assays were performed using either GAST-Fe media [45, 46] or 7H9 media supplemented with 10% Albumin Dextrose Catalase (ADC) [45, 46]. 10 mM stock solutions of the test compounds in DMSO were serially diluted 2-fold, and plates were set up as previously described [47]. Rifampicin at 2xMIC was used as a minimum growth control. Relative fluorescence was measured and used to calculate the MIC as previously reported [22, 48].

### 4.2. Cytotoxicity Analysis

The reduction of 3-[4,5-dimethylthiazol-2-yl]-2,5-diphenyl tetrazolium bromide (MTT) in the presence of living cells was used to evaluate cell viability [49]. 20 mg/mL stock solutions of the test compounds in 10% DMSO were serially diluted in a complete medium with 10-fold dilutions to obtain 6 concentrations. Cells were exposed to the respective test compounds for 44 hours after which MTT was added and cells were incubated at 37°C for a further 4 hours. Plates were analyzed at 540 nm using a spectrophotometer with wells containing untreated cells and growth medium only used to determine 100% survival and 0% survival, respectively. Emetine was used as a positive control, and assays were performed in triplicate. Cell viability was not compromised at the highest concentration of DMSO to which the cells were exposed (data not shown). IC_50_ values were obtained from full dose-response curves, using a nonlinear dose-response curve fitting analysis via GraphPad Prism v.4 software.

### 4.3. Respiratory Reporter Assay

*Mycobacterium tuberculosis* H37Rv ΔcydKO and *Mycobacterium tuberculosis* H37Rv ΔcydKO/QcrBA317T [34, 35] were utilized as described above for the determination of the minimum inhibitory concentration (Section 4.1).

### 4.4. LUX Assays

A modified bioluminescence (LUX) reporter assay was used to investigate cell wall damage and genotoxicity as possible mechanisms of action [37]. A 10 ml culture of the relevant *Mycobacterium tuberculosis* H37Rv strain (PiniB-LUX determining cell wall damage or PrecA-LUX determining DNA damage) was grown to an optical density (OD_600_) of ±0.4. The strain culture was diluted 1 : 10 prior to inoculation of the assay. Two-fold serial dilutions of the test compounds and the assay control drugs were prepared in flat-bottomed 96-well microtiter plates. The relevant diluted *M. tuberculosis* reporter culture was added to each well to a final volume of 100 *μ*l per well. SQ109 and levofloxacin were used as a positive and negative control, respectively, in the P*iniB*-LUX assay, whereas levofloxacin and ethambutol were utilized as positive and negative control, respectively, in the P*recA*-LUX assay.

Raw luminescent data (Relative Luminescence Units) were acquired using a SpecraMax i3x Plate reader (Molecular Devices Corporation, 1311 Orleans Drive, Sunnyvale, California 94089). Data were corrected for background luminescence using Softmax® Pro 6 software (Version 6.5.1, Serial no. 1278552768867612530), Molecular Devices Corporation, 1311 Orleans Drive, Sunnyvale, California 94089. Data were converted to 2-colour 2-dimensional heat maps using Microsoft® Excel® for Mac 2011, Version 14.6.5 (160527), latest installed update 14.6.5.

### 4.5. Synergism Assays

Possible drug interactions of the various test compounds and spectinomycin were investigated utilizing a slightly modified standard two-dimensional (2D) checkerboard assay [42]. Serially diluted drugs were dispensed along the *x*-axis and *y*-axis of 96-well microtiter plates, respectively, at a starting concentration 100 times the final concentration. The assays were performed as previously described [42], and results were reported as a percentage growth inhibition at various drug concentration combinations.

## Figures and Tables

**Table 1:** Antimycobacterial activity and cytotoxicity data.

Compound	MIC_99_	IC_50_ CHO
	7H9-ADC		GAST-Fe
**1** [23]	>125	>125	>10^#^
**2** [24]	>125	>125	>322
**3** [19]	82.20	82.8	119.00
4 [23]	>125	>125	>254
**5** [23]	125	80.9	84.80
**6** [23]	125	>125	>254
**7** [18]	68.70	>125	9.54
**8** [25]	>125	>125	Nd
**9** [26]	>125	>125	>414
**10** [26]	>125	>125	>349
**11** [27]	50.00	42.8	7.40
**12** [26]	>125	>125	>386
**13** [28]	>125	>125	>100^+^
**14** [25]	9.60	18.8	8.01
**15** [25]	13.90	15.2	8.20
**16**	>125	>125	>100^+^
Rifampicin	0.0149	0.0274	Nd
Emetine	Nd	nd	0.061

TB : MIC_99_ 7H9 (*μ*M) in *M. tuberculosis* H37Rv:*gfp*. TB : MIC_99_ GAST-Fe (*μ*M) in *M. tuberculosis* H37Rv:*gfp*. CHO : Chinese hamster ovarian cell IC_50_ (*μ*M) as indication of cytotoxicity [18, 19]. nd: not determined. ^#^highest concentration measured.  ^+^CellTiter-Glo® luminescent cell viability assay [28].

## Data Availability

Data are available on request from the corresponding author through e-mail (ekapp@uwc.ac.za).
